# How CMAS infiltration degrades grain boundaries in thermal barrier ceramics: dual pathways to embrittlement

**DOI:** 10.1038/s41529-026-00843-3

**Published:** 2026-07-17

**Authors:** Yang Liu, Craig J. Williams, Gyaneshwara Brewster, Alison Pawley, Ping Xiao, Ying Chen

**Affiliations:** 1 Department of Materials, The University of Manchester, Manchester, UK; 2 Henry Royce Institute, The University of Manchester, Manchester, UK; 3 Department of Earth and Environmental Sciences, The University of Manchester, Manchester, UK; 4 Rolls-Royce plc, Derby, UK

**Keywords:** Engineering, Materials science

## Abstract

Thermal barrier coatings used in aeroengines are vulnerable to calcia–magnesia–alumino–silicate (CMAS) attack, which infiltrates through pores and grain boundaries (GBs), accelerating coating degradation. However, the influence of CMAS on GB fracture resistance remains insufficiently understood. Here, we combine high-resolution characterization, micromechanical testing, and density functional theory (DFT) calculations to elucidate how infiltrated CMAS degrades fracture toughness and alters fracture behaviour in yttria-stabilized zirconia (YSZ), a model thermal barrier ceramic. Two distinct GB types are identified in CMAS-infiltrated layers: (i) GBs containing nanoscale glassy CMAS films near the surface, and (ii) GBs enriched with CMAS-derived cations (Al³⁺ and Si⁴⁺) but without discernible glassy phases at greater depths. Micromechanical tests reveal a fracture toughness gradient that increases from the surface inward, accompanied by a transition from intergranular to transgranular fracture. When GBs are filled with glassy CMAS films, cracks preferentially propagate through the intergranular CMAS layers due to its low intrinsic toughness, leading to pronounced GB embrittlement. However, an unexpected yet important finding is that GBs doped with Al³⁺ and Si⁴⁺ also exhibit significant embrittlement even in the absence of continuous glassy CMAS films. DFT calculations show that the incorporation of Al³⁺ and Si⁴⁺ into GBs weakens Zr-O bonds along potential fracture paths through site-specific substitution or interstitial occupation. This bond-weakening effect—independent of whether Al³⁺ or Si⁴⁺ acts alone or together—increases GB energy while reducing separation energy, thereby lowering fracture toughness with increasing impurity concentration. The study provides direct atomistic insight into CMAS-induced GB embrittlement mechanisms in thermal barrier ceramics.

## Introduction

Thermal barrier coatings (TBCs) made of yttria stabilized zirconia (YSZ) are widely used in aircraft engines to protect hot-section components from high-temperature and environmental degradation^1–4^. However, they are vulnerable to attack by molten CMAS, which infiltrates the porous coating structure at high temperatures and, upon solidifying during cooling, reduces the coating strain tolerance^5,6^, thereby accelerating failure^7–9^. In addition to this well-established degradation pathway, numerous studies have reported a distinct and equally critical feature of CMAS attack: preferential infiltration along GBs^10–15^. This phenomenon is particularly pronounced in thermally sprayed TBCs, such as those fabricated by atmospheric plasma spray (APS) or suspension plasma spray (SPS), owing to their fine grain size and high GB density^16^. Compared to the bulk lattice, GBs play a dual role in ceramics: they act as structural connections between grains and as chemically active interfaces with higher diffusivity^17^ and lower atomic coordination^18^. Previous studies proposed several mechanisms to explain the tendency of CMAS to concentrate at GBs^19,20^. When exposed to molten CMAS, YSZ GBs can serve as preferential diffusion pathways for constituent elements (Si, Ca, Al), and as reactive sites for the formation of siliceous or complex impurity^21^, silicate phases^22^, and oxide-silicon interfaces^23^. These reactions degrade GBs via two main mechanisms: structural effects, involving changes in atomic arrangement, and chemical effects, involving introduction of CMAS or CMAS-related impurities and changes in electronic structure. Both modify the local chemistry, bonding environment, and structural integrity of the GB^24,25^. In most cases, the reaction products are glassy and amorphous^26^, with a relatively low fracture toughness (≈0.75 MPa√m)^27^, which could reduce the fracture resistance of the boundary region.

Despite the repeated observation of CMAS GB infiltration in both laboratory tests and field-exposed components, the impact of CMAS on the mechanical properties of these GBs has not been thoroughly investigated, particularly with respect to accurately quantifying its effect on GB fracture toughness and behavior. This challenge arises from the inherently complex, porous microstructure and multi-scale grain size distribution of typical TBCs^28^, as well as the graded nature of CMAS infiltration^29^. This gap in knowledge is significant because the GB network often governs the overall fracture behavior of polycrystalline YSZ, including the splats in APS coatings. If CMAS infiltration preferentially weakens GBs, then the onset of coating failure may be controlled as much by intergranular fracture mechanics as by bulk chemical degradation. Establishing a quantitative relationship between intergranular CMAS content and GB fracture resistance would therefore provide a crucial link between microstructural chemistry and macroscopic reliability.

In this work, we develop a synergistic experimental and computational approach to quantitatively evaluate the impact of CMAS infiltration on the fracture toughness and behavior of model YSZ ceramics, providing direct insight into the role of intergranular CMAS in GB embrittlement in TBC ceramics. Different from our previous work based on co-sintered YSZ–CMAS model ceramics^25^, the present study uses molten CMAS infiltration to generate a depth-dependent degradation gradient, allowing the relationship between infiltration depth, GB chemistry, fracture mode and local fracture toughness to be examined directly. Dense YSZ ceramics were first sintered and then infiltrated by molten CMAS to produce a gradient microstructure, simulating the progressive CMAS penetration process in TBCs. The use of dense YSZ ceramics provides a simplified model microstructure and ensures that CMAS infiltration occurs predominantly along GBs. After that, we studied the GB chemistry, fracture toughness, and fracture mode of CMAS-infiltrated YSZ ceramics at different depths using high-resolution characterizations and microcantilever beam bending tests. To gain atomistic insight into how CMAS impurities degrade GB fracture resistance, first-principles calculations based on density functional theory (DFT) were performed, which were not included in our previous co-sintered YSZ–CMAS study^25^. The segregation energies of CMAS impurities at different substitutional and interstitial sites within the GB core were computed to evaluate their impact on GB stability and their site preferences. The GB bonding characteristics were analyzed by the Crystal Orbital Hamilton Population (COHP)^30^ with ICOHP quantifying bond strength and the partial density of states (PDOS). The GB fracture resistance was further estimated by GB separation energy, determined from a free surface (FS) model, thereby linking impurity site preference, local bonding modification and GB separation resistance.

## Results and discussion

### Microstructure

**Fig. 1:**
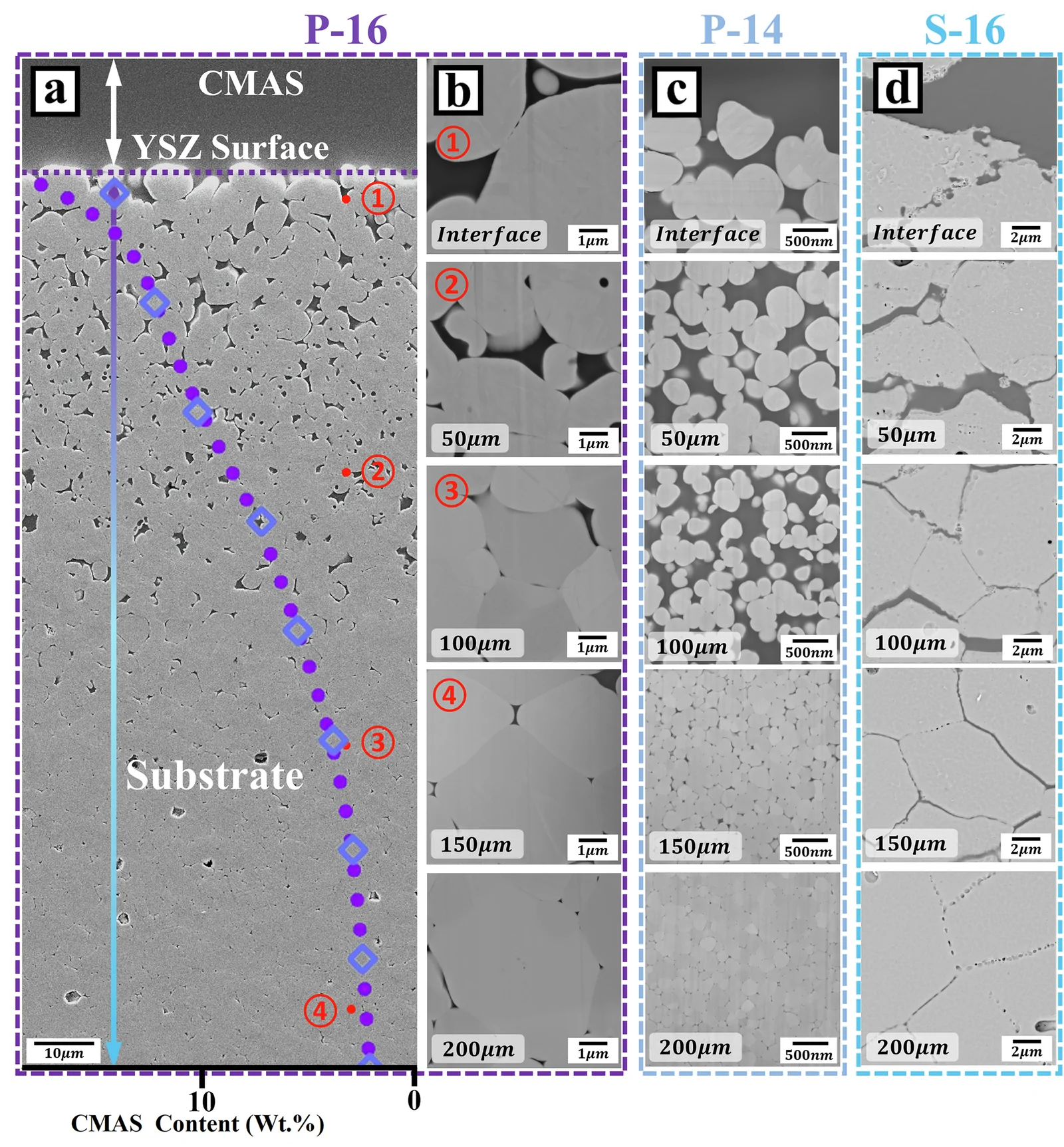
Cross-sectional microstructures of YSZ ceramics after CMAS infiltration at 1300 °C for 2 h. **a** Overview of P-16 after CMAS infiltration. The purple dotted line indicates the depth-dependent CMAS content profile, with the corresponding CMAS content percentage shown on the lower x-axis; **b** Magnified BSE images of local microstructures at different depths from the surface (0, 50, 100, 150 and 200 μm) in (**a**); **c**, **d** BSE images of local microstructures at different depths from the surface (0, 50, 100, 150 and 200 μm) in P-14 and S-16, respectively, after CMAS infiltration. Note that the scale bars vary across image groups (**b**–**d**).

The average grain sizes of the ceramic pellets, determined by image analysis of polished cross-sections without CMAS infiltration, are ~0.24 μm for P-14, ~3.11 μm for P-16 and ~8.94 μm for S-16, respectively. Figure 1a shows that the cross-section of P-16 after CMAS infiltration exhibits a gradient structure through the thickness. The overlaid CMAS content profile in Fig. 1a was estimated from image analysis of SEM-visible CMAS pockets and intergranular CMAS features, providing a quantitative indication of the decrease in resolvable CMAS content with depth. The CMAS/YSZ interface is clear and many spherical reprecipitated YSZ grains are observed in the heavily infiltrated surface region. The detailed microstructure of CMAS infiltrated P-16 at different depths (Fig. 1b) shows that YSZ GBs (including boundaries of reprecipitated YSZ grains) are infiltrated and separated by CMAS, leading to the formation of intergranular CMAS films and SEM-visible CMAS pockets at triple junctions. With increasing infiltration depth, the size of CMAS pockets at triple junctions decreases rapidly (Fig. 1b), and the glassy intergranular films become progressively less pronounced and eventually becoming difficult to detect under SEM. Compared to P-16, the P-14 sample with smaller grains exhibits deeper SEM-visible CMAS penetration (~44% more, Fig. 1c), whereas the S-16 sample with larger grains shows shallower SEM-visible CMAS infiltration (~37% less, Fig. 1d). Collectively, these findings demonstrate that YSZ GBs serve as preferential and effective pathways for CMAS infiltration, consistent with previous reports^31–35^.

**Fig. 2:**
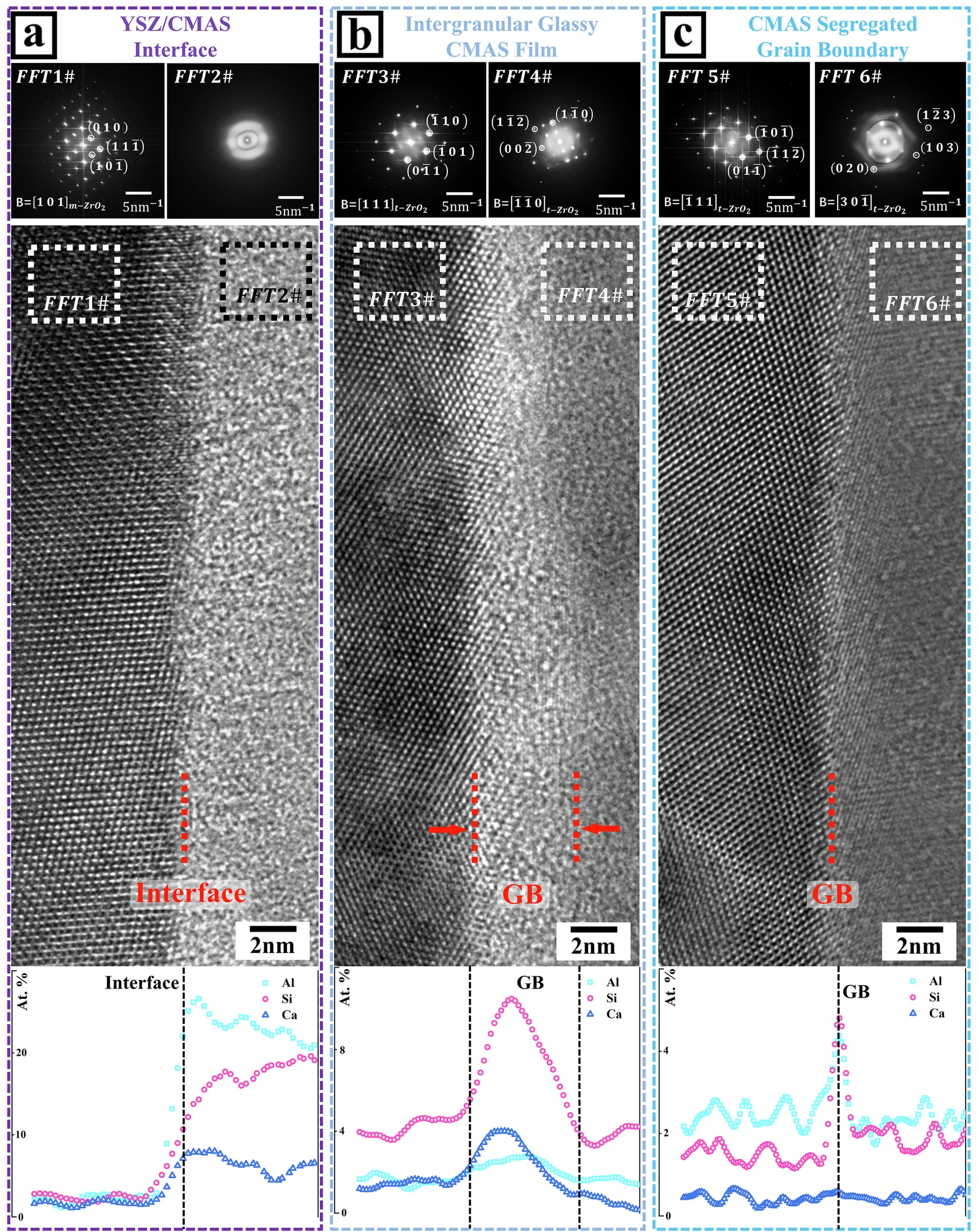
High-resolution TEM (HRTEM), local fast Fourier transform (FFT) and EDS-line characterizations of GBs in CMAS-infiltrated P-16. **a** At the YSZ/CMAS interface; **b** At a depth of 50 μm from the YSZ/CMAS interface; **c** At a depth of 200 μm from the YSZ/CMAS interface: no intergranular glassy film is present, but the GB is segregated with Si and Al derived from CMAS. It should be noted that these characteristic GBs are present at all depths; the variation lies in their relative proportions. Essentially, most GBs near the surface are occupied by CMAS films, whereas those in deeper regions typically lack CMAS films but are enriched with Si and Al.

As described above and shown in Fig. 1, most GBs at depths greater than ~100 μm lacked distinct SEM-visible intergranular CMAS films or pockets. To clarify whether CMAS-derived species are still present at GBs beyond this depth, the composition and structure of GBs in CMAS infiltrated P-16 were analyzed by high-resolution TEM, and the results are shown in Fig. 2. In the region close to the surface, YSZ grains are clearly separated by a relatively thick layer of CMAS (Fig. 2a). The CMAS/YSZ interface appears well-defined, with no discernible transition layer in between. The CMAS remains in an amorphous state without any crystalline phases, but the adjoining reprecipitated YSZ grains have transformed from the original tetragonal to monoclinic structure (indicated by FFT2# in Fig. 2a). At depths of ~50 μm and ~200 μm, two distinct types of GB are observed. The first type exhibits a clear but thinner amorphous CMAS intergranular film (Fig. 2b), compared to that in the surface region (Fig. 2a). EDS line analysis confirms the segregation of Si, Al, and Ca at the GB, aligning with the major cations present in CMAS. In contrast, the second type lacks a discernible glassy CMAS film even at the atomic scale, as illustrated in Fig. 2c. However, Si and Al segregation, but not Ca segregation, was still identified at the GB, suggesting that CMAS-derived species migrated into GBs that appeared unaffected in SEM images despite the absence of a glassy CMAS film. The absence of Ca segregation at the GBs is likely attributed to both the diminishing SEM-visible CMAS content with the progression of the infiltration front and the dissolution of CaO into YSZ at high temperatures, given its high solubility in ZrO_2_^36,37^. The absence of an intergranular glassy film can be attributed to the lack of Ca, which is typically required to promote the formation of a glassy phase in CMAS systems^38^. For both types of GBs, the adjacent YSZ grains remain in the tetragonal phase, indicating that the presence of CMAS at these locations does not induce a phase transformation in YSZ, an observation further confirmed by local FFT. Nevertheless, the presence of intergranular CMAS, either as glassy films or CMAS-derived impurities (Al and Si), alters the local chemical environment at YSZ GBs, an effect that is expected to influence the mechanical properties of the ceramics.

### Fracture features and fracture toughness

**Fig. 3:**
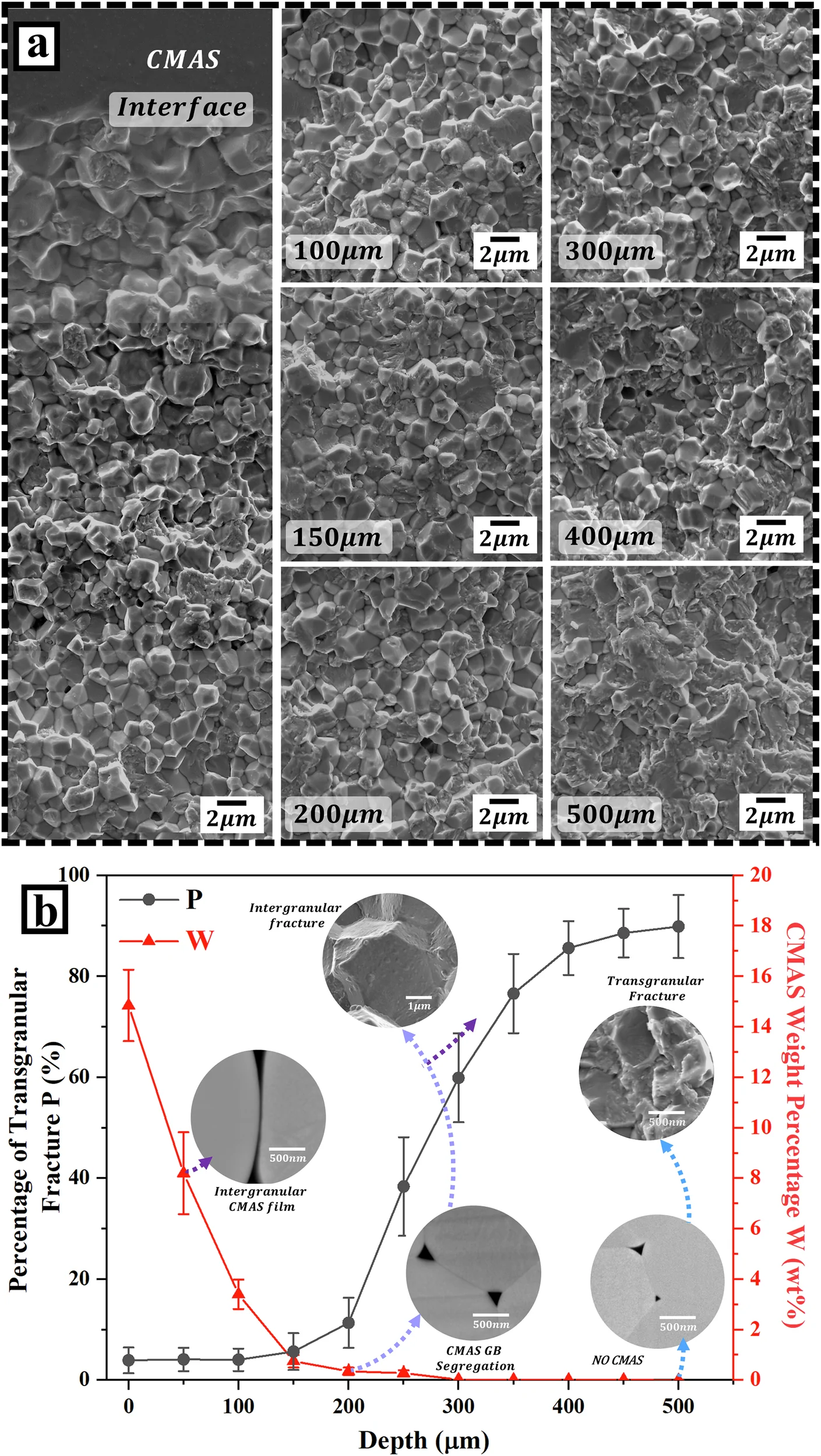
Analysis of the fractured cross-section in CMAS-infiltrated P-16. **a** SEM images at various depths; **b** CMAS weight percentage (red line) and proportion of transgranular fracture (black line) as a function of depth. The two quantities were estimated through quantitative analysis of polished and fractured cross-sections, respectively, at various depths using ImageJ. Only CMAS pockets located at triple junctions and intergranular CMAS films identifiable under SEM were considered in calculating the CMAS weight percentage. The CMAS density used in the calculation was taken to be 2.69 g/cm^3^^27^. Six SEM images were analyzed at each depth to obtain statistically representative average values.

**Fig. 4:**
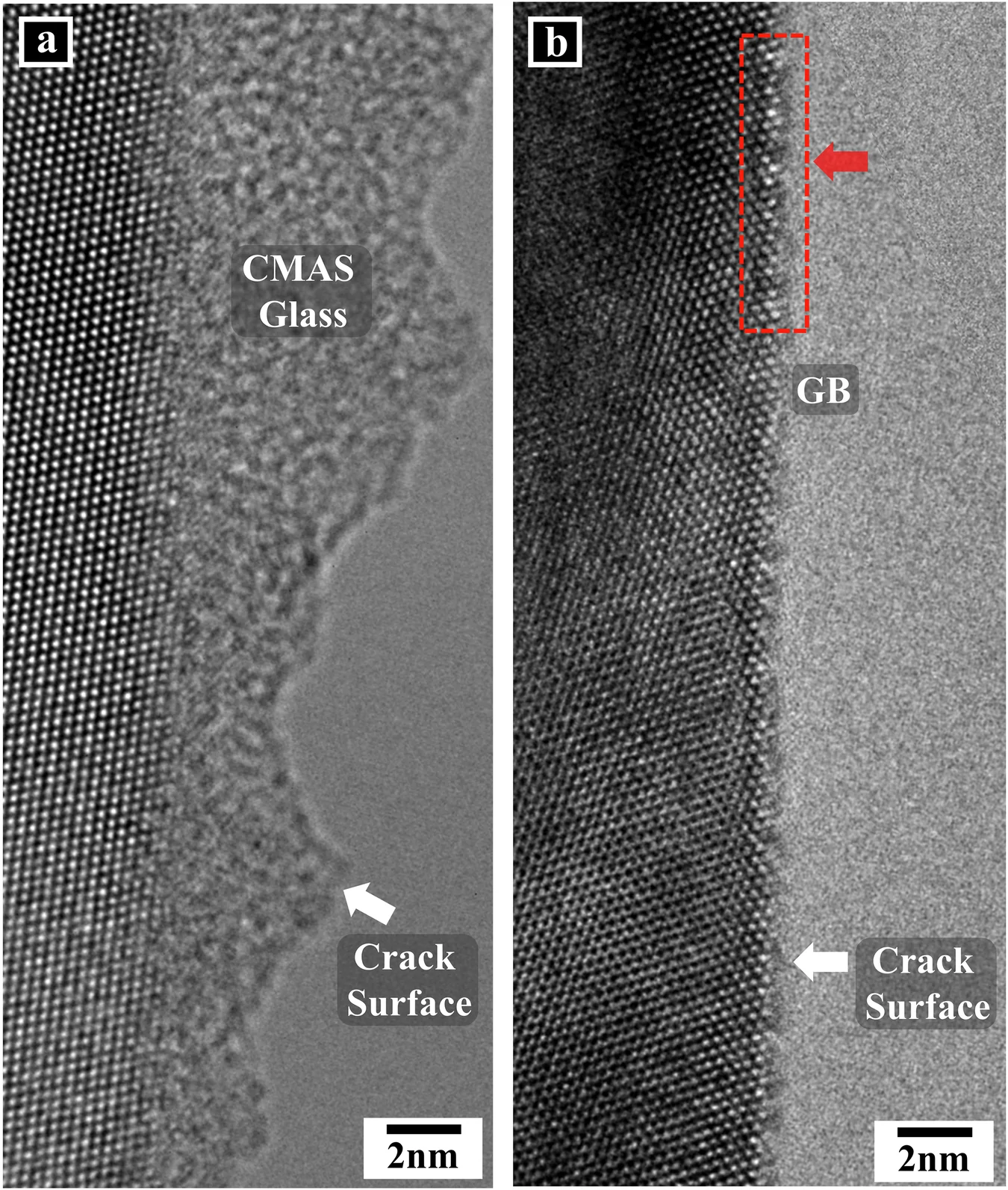
HRTEM images showing crack paths in CMAS-infiltrated P-16. **a** Crack propagation through an intergranular CMAS film and **b** Crack propagation along a glass-free GB. The brighter contrast along the GB represents the light cations such as Si^4+^ and Al^3+^ incorporated into the GB from CMAS.

**Fig. 5:**
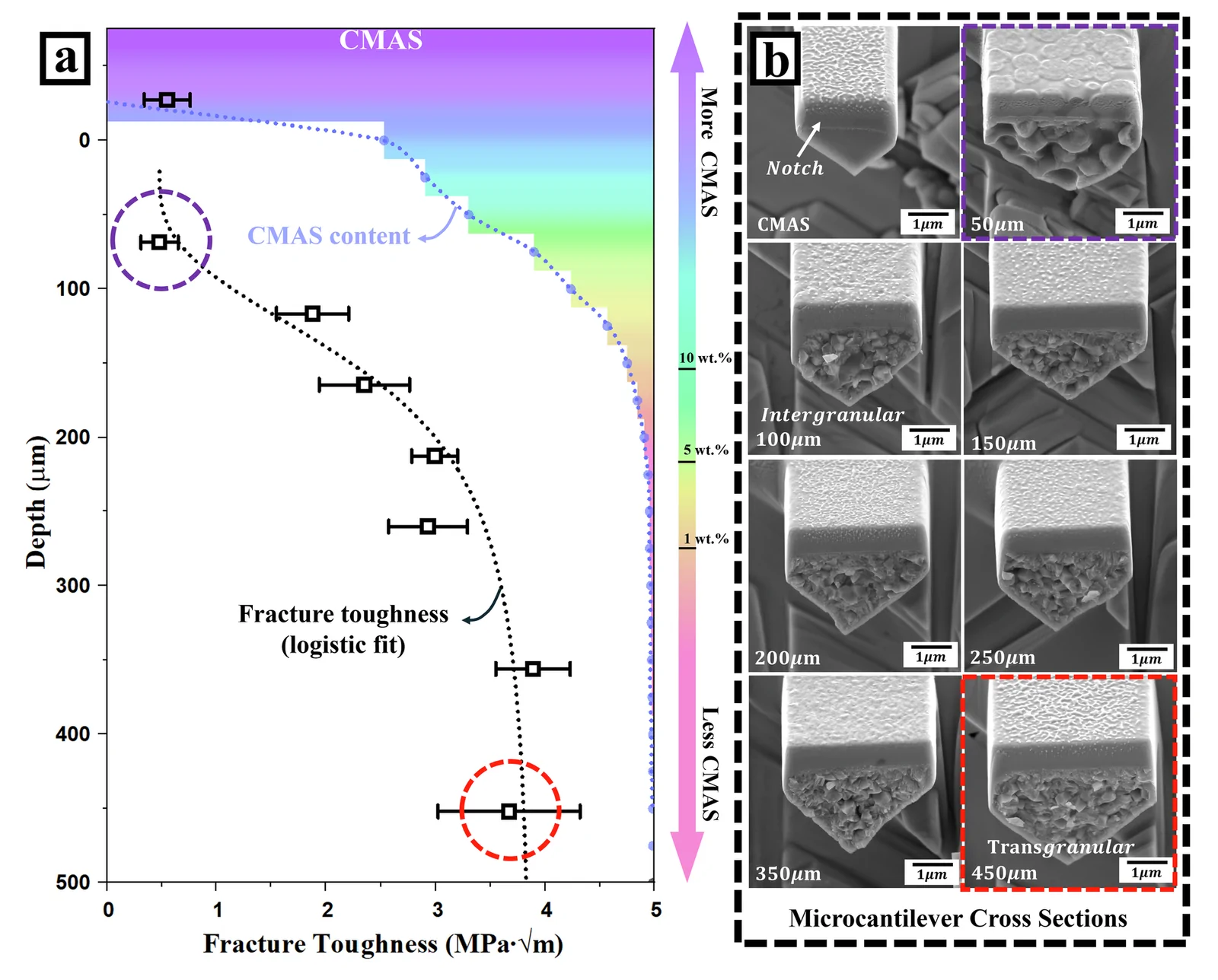
Evolution of fracture toughness and fracture mode as a function of depth in CMAS-infiltrated P-14. **a** Fracture toughness values measured at different depths from the YSZ/CMAS interface; the colored bar represents the CMAS content at the corresponding depth. **b** Fracture mode transition revealed by fractured cross-sections of microcantilever beams at different depths, showing a shift from intergranular fracture near the YSZ/CMAS interface to transgranular fracture at greater depths.

To establish a quantitative relationship between the fracture behavior and the intergranular CMAS content, CMAS-infiltrated P-16 was fractured along the cross-section using a three-point bending setup, with the CMAS-infiltrated side positioned under tension. P-16 was selected for this analysis because its intermediate grain size allows the depth-dependent transition in fracture morphology to be resolved clearly while still preserving a well-developed CMAS infiltration gradient. The resulting fracture surfaces at various depths (0–500 μm) are shown in Fig. 3a. Overall, there is a transition in fracture mode from intergranular fracture in the outer regions with intergranular CMAS films and/or CMAS-derived impurities to transgranular fracture in the inner pristine YSZ, indicating that CMAS leads to embrittlement at YSZ GBs. From the top CMAS/YSZ interface down to a depth of ~200 μm, fracture is predominantly intergranular. The prevalence of intergranular fracture in these regions is expected, given the presence of the intergranular CMAS film network and the inherently low fracture toughness of glassy CMAS (0.54 MPa√m, Fig. 5a). For the local GB structure examined in Fig. 4a, TEM analysis shows that the crack propagates within the amorphous CMAS-rich intergranular region. It should be noted that the exact crack path in glassy CMAS-containing GBs may depend on the local CMAS/YSZ interfacial structure. In our previous co-sintered YSZ–CMAS ceramics^25^, cracks were observed to propagate along the CMAS/YSZ interface, where a monoclinic YSZ transition layer was present. In the present molten-infiltrated sample, however, the CMAS/YSZ interface in Fig. 4a appears sharp, with a direct transition from YSZ to amorphous CMAS and without an obvious intermediate transition layer. Therefore, both interfacial cracking and cracking through the CMAS-rich phase can occur in CMAS-modified GBs, depending on the local GB structure. Consequently, the fracture of the intergranular CMAS phase dominates the energy dissipation in the surface region. This also suggests that the fracture toughness of the surface region may be comparable to that of CMAS itself, which will be verified in our follow-up fracture tests.

As one moves further into the inner part of the ceramic, the fracture becomes a mixture of transgranular and intergranular modes, with the proportion of transgranular fracture gradually increasing. At a depth of ~500 μm and beyond, the fracture mode becomes almost exclusively transgranular. Statistical evaluation based on quantitative image analysis of fractured and polished cross-sections reveals that the percentage of transgranular fracture is inversely proportional to the CMAS content along the depth (<500 μm), as plotted in Fig. 3b. Apart from the predominant intergranular fracture pattern in the surface regions, a striking feature shown in Fig. 3b is that intergranular fracture still accounts for 88% to 15% of the fracture morphology between depths of 200 μm and 400 μm where intergranular glassy CMAS films are typically not detected (Fig. 1 and Fig. 2c) and the estimated CMAS content is extremely low (<1 wt%). The observation has two important implications. First, it suggests that the influence of CMAS can extend beyond 200 μm: CMAS is mainly present as intergranular glassy films in the surface regions, whereas at greater depths it may be manifested primarily as CMAS-derived GB impurities, such as Al and Si, as the amount of SEM-visible CMAS decreases along the advancing infiltration front. Second, not only intergranular glassy CMAS films, but also CMAS-derived impurities (Fig. 2c) can weaken YSZ GBs, contributing to the considerable proportion of intergranular cracking observed at depths beyond 200 μm. HR-TEM analysis of the glass-free GB after fracture (Fig. 4b) shows a smooth and clean morphology with brighter contrast along the exposed GB surface (e.g., the region highlighted in the red box). The brighter contrast is likely attributed to the incorporation of light CMAS cations such as Si^4+^ and Al^3+^ into the GB.

To further quantitatively investigate the influence of intergranular CMAS on fracture toughness, multiple sets of microcantilever beams were milled by FIB at various depths—from the top CMAS layer down to a depth of 450 μm from the ceramic surface—along the cross-sections of CMAS infiltrated P-14. P-14 was selected for micromechanical testing because its small grain size (~0.24 μm) ensures that each fractured microcantilever cross-section contains a sufficient number of grains (~50) to provide statistically representative depth-dependent fracture-toughness and fracture-mode data, whereas the larger-grained P-16 and S-16 samples would make the results more sensitive to individual grain/GB orientation and character. The fracture toughness of the residual CMAS glass was measured as a reference for comparison. Overall, the fracture toughness progressively increases from the surface toward the interior of the ceramic along the direction of CMAS infiltration (Fig. 5a), accompanied by a transition from intergranular fracture to transgranular fracture (Fig. 5b), consistent with the observations made in CMAS-infiltrated P-16 (Fig. 3). The fracture toughness near the surface region (e.g., at a depth of 50 μm) is measured to be 0.48 MPa√m, closely matching that of the residual CMAS glass on the top surface (0.54 MPa√m), as cracking occurs predominantly through the thick CMAS films between the relatively coarse reprecipitated YSZ grains (Fig. 5b). At depths between 100 and 250 μm, the fracture toughness increases by a factor of four to six compared to that of the surface region, although the fractured cross-sections of the microcantilever beams remain primarily intergranular (Fig. 5b). Within this depth range, the intergranular CMAS films become progressively thinner (e.g., Fig. 2), and an increasing number of GBs no longer contain CMAS films but instead show segregation of CMAS-derived impurities such as Al and Si (e.g., Fig. 2). This suggests that as the depth increases further into the interior, fewer GBs are affected by CMAS-derived impurities, and their concentrations gradually decrease to zero. Consequently, the fracture toughness recovers to the intrinsic value of YSZ and fracture becomes predominantly transgranular (Fig. 5b).

### GB embrittlement mechanism by first-principles calculations

Through high-resolution characterizations, we have demonstrated that CMAS infiltration significantly alters GB chemistry of YSZ ceramics by introducing nanoscale intergranular glassy films or CMAS-derived intergranular impurities, including Si^4+^ and Al^3+^. Fractographic and micromechanical analyses have shown that the change of GB chemistry leads to a shift in fracture mode from transgranular to intergranular, accompanied by a substantial reduction in fracture toughness (by as much as 80%). The quantitative relationship established between CMAS content, fracture toughness and fracture mode highlights the detrimental role of intergranular CMAS in degrading the fracture resistance of YSZ ceramics. When present as an intergranular glassy CMAS film, the CMAS-modified GB region provides a preferential fracture path because of the low fracture resistance of the glassy phase and/or its interface with YSZ, thereby embrittling the YSZ GBs. However, this explanation does not apply to the embrittlement of GBs that are doped only with CMAS-derived impurities (Si⁴⁺ and Al³⁺) in the absence of a continuous glassy CMAS film—an unexpected yet important finding of this study. To answer this question, DFT calculations were conducted to understand the atomic-scale origins of Si^4+^ and Al^3+^-induced GB embrittlement.

It should be noted that the DFT calculations use a simplified Σ5(310)/[001] ZrO₂ GB model. This model helps isolate the effects of Si and Al on local GB bonding and separation resistance, but it does not fully represent experimental conditions with various GB characters, Y segregation, oxygen vacancies, residual stresses, and complex CMAS-derived chemistry. Therefore, the calculated GB energy and separation energy are considered qualitative or semi-quantitative indicators of GB stability and cohesion, rather than direct quantitative predictions of the measured fracture toughness. Here, the DFT results are used to provide mechanistic insight into Si/Al-induced weakening of glass-free GBs.

**Fig. 6:**
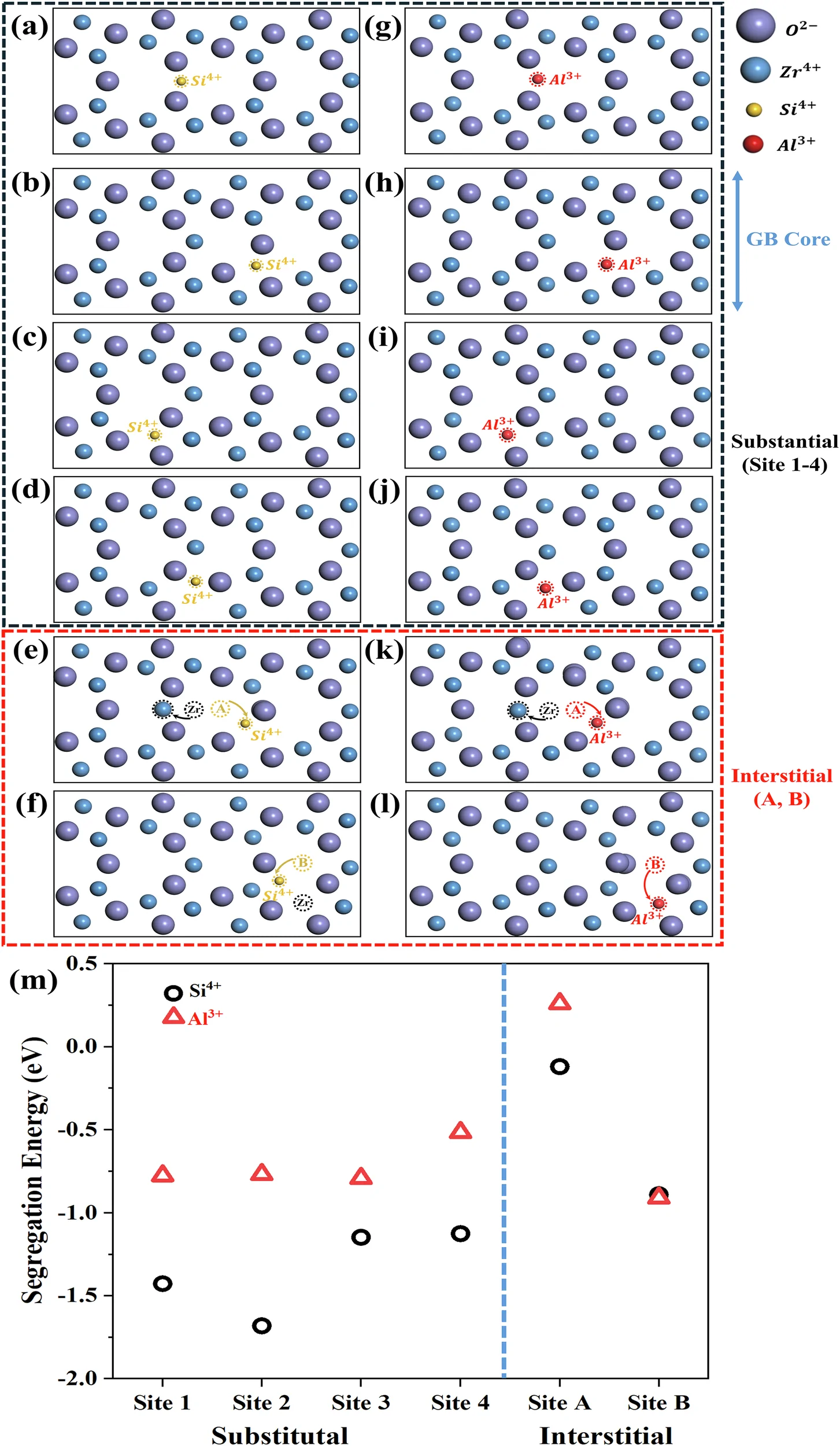
DFT calculations showing the site preference of CMAS-derived impurities (Si⁴⁺ and Al³⁺) at the model Σ5(310)/[001] GB core. **a**–**f** show the relaxed Si-doped configurations, **g**–**l** show the relaxed Al-doped configurations, and **m** summarizes the site-dependent segregation energies of substitutional and interstitial Si⁴⁺ and Al³⁺ cations.

To investigate the embrittlement mechanism associated with the segregation of Si^4+^ and Al^3+^ to the GB, we first examined their possible positions within GB core model by DFT, including four substitutional sites (site 1–4) and two interstitial sites (site A and B), as shown in Fig. 12a. After full relaxation, the structures are shown in Fig. 6a–l, with the resulting trends summarized in Fig. 6m. For the substitutional configurations, as shown in Fig. 6a–d, g–j, both Si^4+^ and Al^3+^ exhibit negative segregation energies at all considered sites, indicating a clear tendency to segregate to the GB, in agreement with experimental observations^25,39^ and DFT calculations^24^. Compared with Si^4+^, Al^3+^ shows almost identical segregation energies across sites 1–4, suggesting weak site preference. In contrast, Si^4+^ displays a significant site-dependent behavior: site 2 is the most favorable substitutional site, with a segregation energy significantly lower than the other three sites. This preference can be attributed to the ability of site 2 to provide a local coordination environment that supports the formation of strong, tetrahedral-like Si-O bonds, a motif that is not equally favorable at the other substitutional sites. Similar site-dependent behavior of four-fold coordinated dopants at Σ5(310) zirconia GBs has been reported in first-principles studies^40^. For the interstitial configurations, site A initially corresponds to an oxygen vacancy with a high positive charge, which repels both Si^4+^ and Al^3+^, resulting in high-energy, unstable structures. As shown in Fig. 6e, k, the Si^4+^ cation initially placed at site A relaxes toward site 2, displacing a neighboring Zr^4+^ cation in the opposite direction. Al exhibits a strong tendency to adopt four-fold oxygen coordination, consistent with X-ray absorption spectroscopy observations of undersized dopant cations in tetragonal and cubic zirconia^41^. As shown in Fig. 6f, l, at site B, which provides more free space relative to site A, structural relaxation drives Al^3+^ toward the vicinity of site 3, forming a more stable configuration with the lowest segregation energy. Similarly, Si^4+^ relaxes near site 2, forming tetrahedrally coordinated Si-O bonds with the O^2-^ anions adjacent to the neighboring Zr^4+^. Interestingly, Si^4+^ does not move toward the previously removed site 3, indicating that site 2 provides a relatively stable local environment that helps lower the total energy of the system.

**Fig. 7:**
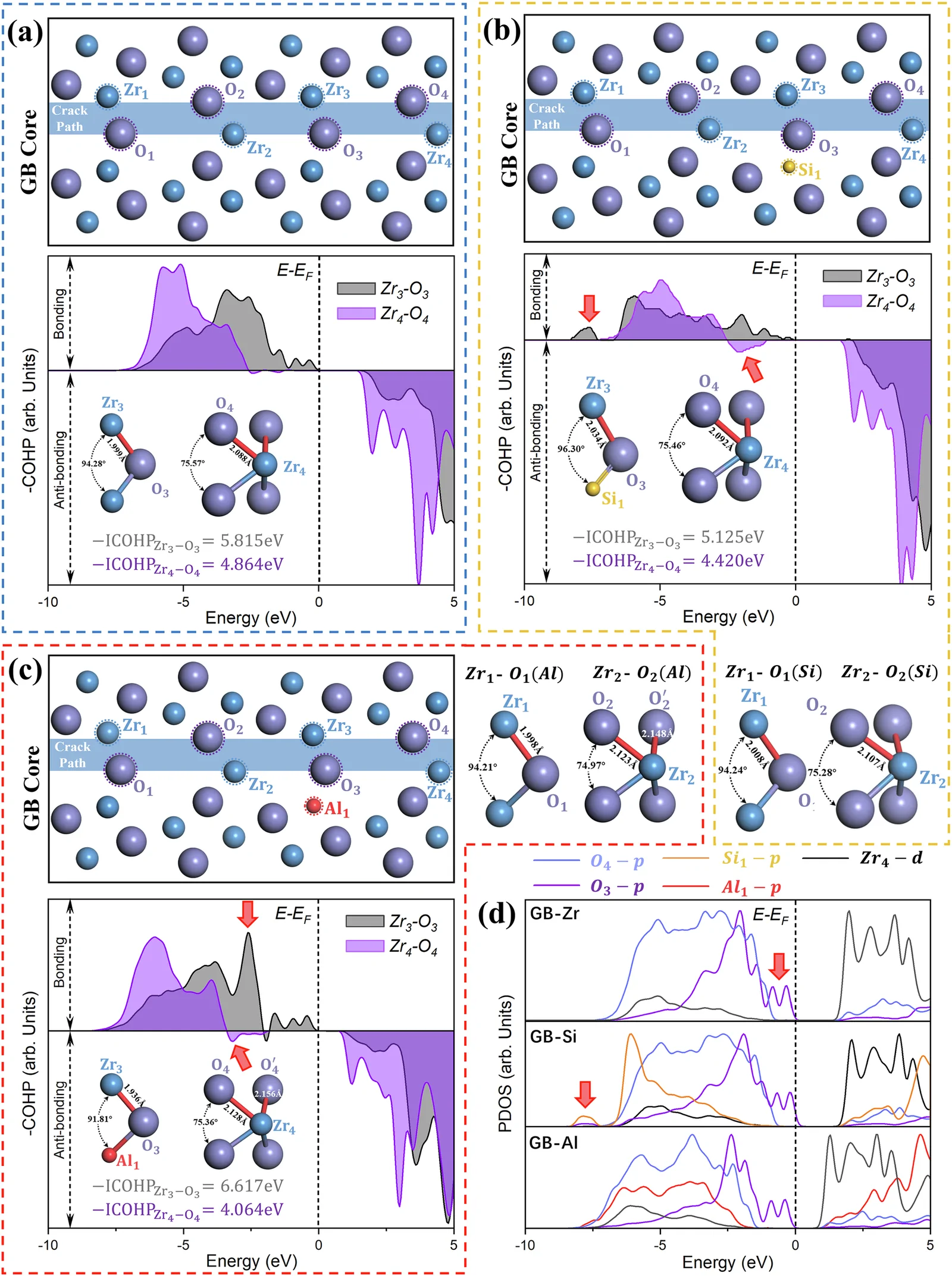
Schematic diagrams of the three GB core models and the corresponding COHP curves for Zr-O bonds along the proposed crack-propagation path (highlighted by the blue region), together with PDOS) analysis. **a** Pristine zirconia GB model, **b** Si⁴⁺ substitution at site 2 in the GB core. **c** Al³⁺ substitution at site 2 in the GB core. **d** PDOS of selected O²⁻, Si⁴⁺, Al³⁺, and Zr⁴⁺ ions in the three GB models, showing the electronic structure variations upon different cation substitutions.

The embrittlement behavior of GB is primarily determined by the nature of chemical bonds across the boundary. We hypothesize that crack propagation in brittle ceramics preferentially follows paths with weaker and fewer bonds, in accordance with the principle of energy minimization. Based on the GB segregation energy calculations described above (Fig. 6m), we selected the site 2 model, which exhibits the lowest system energy, for further investigation. As illustrated in Fig. 7a–c, the blue region indicates the proposed crack-propagation path containing the fewest Zr-O bonds, denoted as Zrᵢ-Oᵢ (*i* = 1–4). In addition, to investigate Zr-O bonding and antibonding interactions along the crack propagation path at the GB core, we calculated the COHP and the corresponding integrated COHP (ICOHP) using the LOBSTER program. Bonding states are indicated by negative COHP values below the Fermi level (E-E_F_ = 0 eV), while antibonding states correspond to positive values (Fig. 7a–c). The integrated form, ICOHP, is an indicator of bond strength—the more negative the ICOHP value, the stronger and more stable the corresponding chemical bond. For clarity, we plot -COHP and -ICOHP, so that bonding interactions appear as positive values.

In the undoped GB core (Fig. 7a), shorter Zr₃-O₃ (1.999 Å) bonds exhibit larger -ICOHP values and a broader bonding energy range (−7 to 0 eV) than the longer Zr₄-O₄ bonds (2.088 Å), indicating stronger orbital hybridization and higher bond strength. As shown in Fig. 7b, when Si⁴⁺ is introduced at site 2, all Zr-O bonds slightly elongate (by up to 1.75%), and the -ICOHP values of Zr₃-O₃ and Zr₄-O₄ decrease by 11.9% and 9.1%, respectively, revealing weakened bonding. The PDOS (Fig. 7d) shows new hybridization between Si₁-p and O₃-p orbitals, which perturbs local bonding but does not strengthen it; instead, the emergence of antibonding states near -3 eV indicates reduced stability. In contrast, as shown in Fig. 7c, Al³⁺ substitution strengthens the nearby Zr₃-O₃ bond (shortened by 3.15%), but weakens the adjacent Zr₄-O₄ bond (lengthened by 1.92%, -ICOHP ↓ 0.8 eV). PDOS (Fig. 7d) analysis suggests predominantly ionic bonding with limited orbital overlap between O-p, Zr-d, and Al-p states, implying that Al doping modifies local charge distribution rather than enhancing covalency. Overall, despite local strengthening, Al³⁺ reduces the average -ICOHP along the fracture path by 0.40 eV, indicating a net loss of GB strength.

**Fig. 8:**
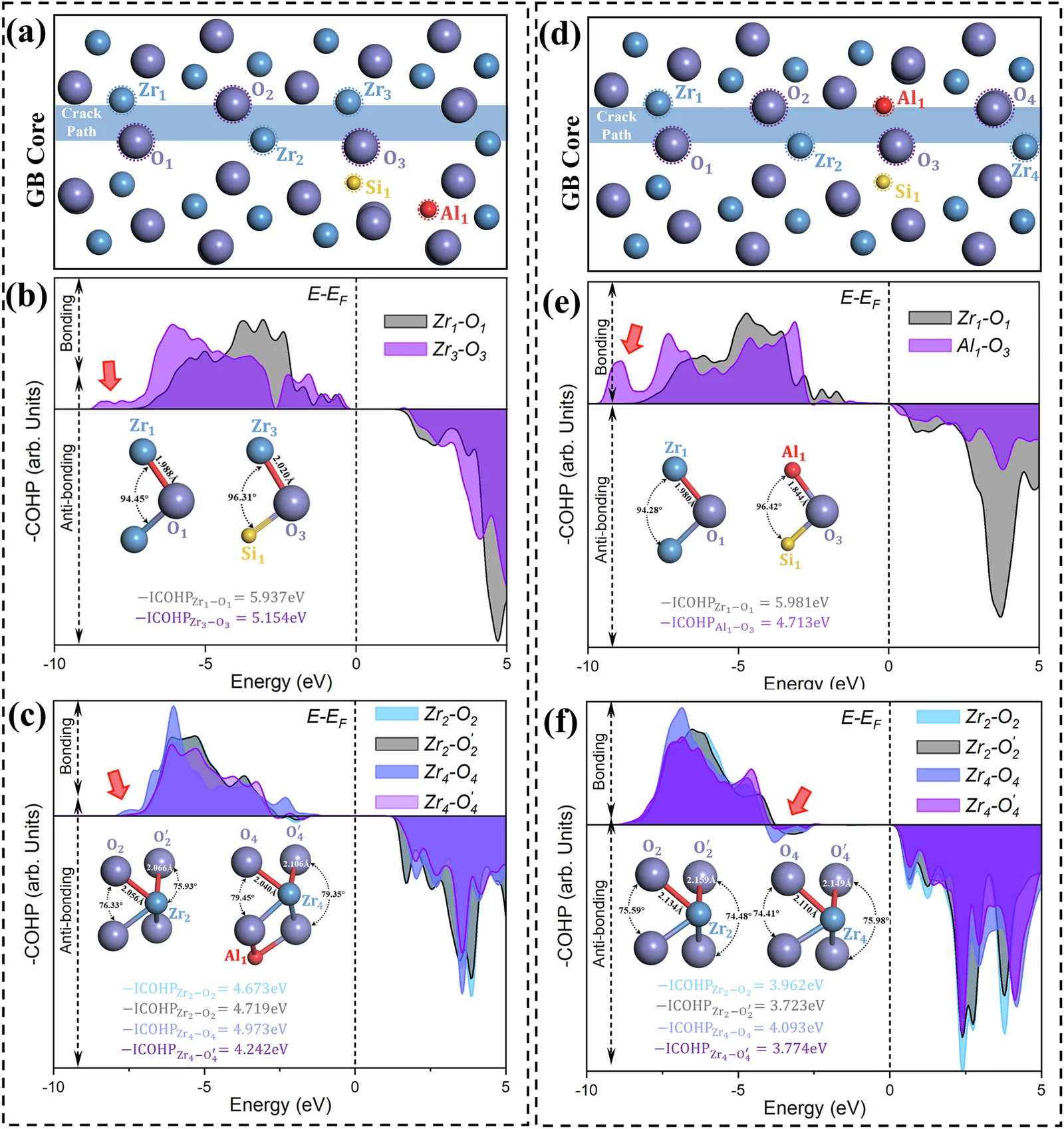
Schematic diagrams of the two Si/Al co-substituted GB core models and the corresponding COHP curves for Zr-O and Al-O bonds along the proposed crack-propagation path (highlighted by the blue region). **a** Si⁴⁺ and Al³⁺ located on the same side of the GB, at sites 2 and 3, respectively. **b**, **c** Corresponding COHP curves for Zr-O bonds and Al-O bonds, respectively, for the same-side substitution model. **d** Si⁴⁺ and Al³⁺ located on opposite sides of the GB, both at site 2. **e**, **f** Corresponding COHP curves for Zr-O bonds and Al-O bonds, respectively, for the opposite-side substitution model.

The above analysis clarifies how single Si⁴⁺ and Al³⁺ dopants weaken GB. To further explore their combined effects, two co-doped GB models were constructed (Fig. 8a, d). When Si⁴⁺ and Al³⁺ occupy adjacent sites on the same side of the GB (Fig. 8a–c), the Zr₁-O₁ and Zr₃-O₃ bonds are strengthened under the influence of Al³⁺, particularly the Zr₃-O₃ bond near Si⁴⁺, whose -ICOHP increases by 16.6% and 6.0% compared with the single-Si-doped and undoped GBs, respectively. Although some neighboring bonds (e.g., Zr₂-O₂, Zr₂-O′₂, Zr₄-O′₄) are slightly weakened, the average -ICOHP along the fracture path decreases by only 0.23 eV relative to pure zirconia—nearly half the reduction observed in the single-Al-doped case. This suggests that same-side Si-Al co-doping exerts only a minor weakening effect on GB cohesion. In contrast, when Si⁴⁺ and Al³⁺ occupy equivalent sites on opposite sides of the GB (Fig. 8d–f), the Zr-O bonds associated with Zr₂ and Zr₄ show a ~20% decrease in -ICOHP compared with the undoped GB, leading to an overall reduction of 0.81 eV in average -ICOHP. This indicates that opposite-side substitution significantly deteriorates GB strength.

In summary, COHP and ICOHP analyses reveal that zirconia GB embrittlement arises from variations in Zr-O bond strength along the crack path. Si⁴⁺ doping weakens bonding by elongating Zr-O bonds and disrupting orbital hybridization, whereas Al³⁺ doping induces charge redistribution that locally strengthens but overall weakens cohesion. When both dopants coexist, the extent of weakening strongly depends on their relative positions—same-side co-doping causes limited degradation, while opposite-side substitution markedly reduces GB strength.

The calculations above revealed the atomistic mechanisms of GB embrittlement associated with the segregation of Si⁴⁺ and Al³⁺ cations. In this section, we further use DFT calculations to examine how CMAS impurity type and configuration affect GB thermodynamic stability and separation resistance and compare these calculated trends with the experimental fracture-toughness evolution. According to Griffith’s fracture theory, the resistance to crack propagation is directly related to the energy required to create new surfaces. Therefore, to evaluate separation resistance of CMAS impurity-doped GBs, we employed relaxed surface slab models derived from the GB configurations. This approach allows the fracture process to be represented as the separation of a GB into two free surfaces, linking surface energy to GB cohesion. As illustrated in Fig. 12b, the doped Si^4+^ and Al^3+^ were placed on the *Surface_down* side, while the opposite *Surface_up* side was kept clean. This configuration ensures that the upper surfaces of all models remain consistent and simplifies the subsequent calculations.

Here, GB energy and separation energy have different physical meanings. GB energy, calculated using Eq. (9), describes the thermodynamic stability of the boundary, with a higher value indicating a less stable GB. In contrast, separation energy, calculated using Eq. (10), represents the work required to cleave the GB into two free surfaces, and is therefore more directly related to GB cohesion and intergranular fracture resistance.

**Fig. 9:**
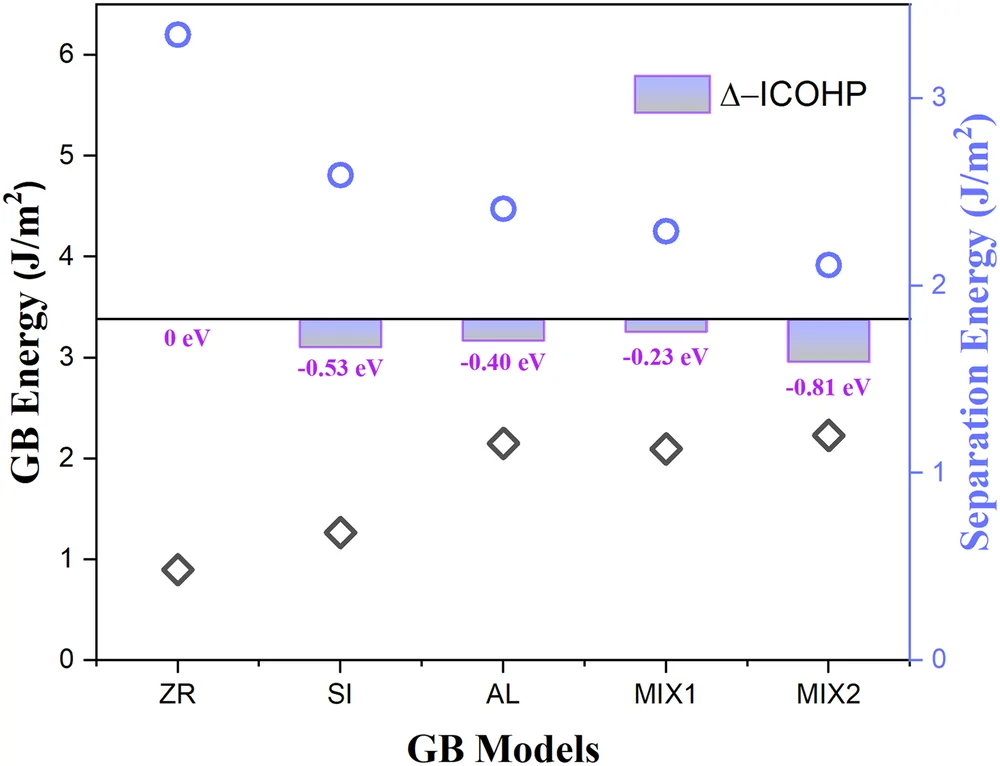
GB energies (black diamonds, left axis), separation energies from the surface model (blue circles, right axis), and changes in average -ICOHP along the fracture path relative to the pure zirconia GB (purple columns) for five GB models: ZR = zirconia GB, Si = Si⁴⁺-doped GB, Al = Al³⁺-doped GB, MIX1 = Si⁴⁺ and Al³⁺ co-doped GB on the same side, and MIX2 = Si⁴⁺ and Al³⁺ co-doped GB on opposite sides. GB energy describes the thermodynamic stability of the boundary, whereas separation energy represents the work required to cleave the GB into two free surfaces and is more directly related to GB cohesion.

In addition, the difference in -ICOHP (ΔICOHP) was determined by subtracting the average -ICOHP value of the pure zirconia GB from the average -ICOHP value along the fracture path of each doped model (Figs. 7 and 8). ΔICOHP is used here as a local bonding descriptor to evaluate bond weakening along the potential fracture path. The results are summarized in Fig. 9, where the samples are labeled as follows: **ZR**—pure zirconia GB; **Si**—Si-doped GB; **Al**—Al-doped GB; **MIX1**—Si and Al co-doped on the same side; and **MIX2**—Si and Al co-doped on opposite sides.

The doping of Si^4+^ and Al^3+^ at the GB leads to a decrease in GB separation energy and an increase in GB energy. This combined trend indicates that CMAS-derived impurities both destabilize the GB thermodynamically and reduce the energy required for GB separation, thereby supporting the GB embrittlement mechanism. The increase in GB energy can be attributed to the significant size mismatch between the dopants and the host cations: the ionic radii of Al³⁺ (67.5 pm) and Si⁴⁺ (54 pm) are substantially smaller than that of Zr⁴⁺ (86 pm)^42^, which increases the pre-existing strain energy and further lowers the barrier for crack initiation and propagation along GBs. While ΔICOHP values quantify local bond strength along the crack path, they do not directly correspond to macroscopic GB energy or separation energy, which also includes elastic and structural contributions. Among the doped models, Si⁴⁺ doping causes the smallest reduction in GB strength, whereas opposite-side Si^4+^ and Al^3+^ co-doping exhibits the lowest separation energy, indicating the most pronounced weakening. Overall, the extent of GB degradation depends strongly on dopant type, site, and configuration.

The GB separation energy (${E}_{\mathrm{sep}}$) represents the theoretical work required to cleave the interface into two free surfaces. A higher separation energy generally indicates stronger interfacial bonding, and thus a higher resistance to intergranular fracture. For brittle ceramics under plane stress, ${K}_{\mathrm{IC}}$ can be qualitatively correlated with ${E}_{\mathrm{sep}}$ according to the Griffith criterion,

1 $${K}_{{IC}}=\sqrt{2E{E}_{{sep}}}$$

Based on the elastic constants of cubic zirconia reported by Hayakawa et al. (C_11_ = 423.7 GPa, C12 = 75.3 GPa, C44 = 47.3 GPa)^43^, the Young’s modulus along the [310] direction was calculated to be 258 GPa using the standard orientation-dependent elastic modulus relationship for cubic crystals, given by:

2 $$\frac{1}{{E}_{[{hkl}]}}={s}_{11}-2\left({s}_{11}-{s}_{12}-\frac{1}{2}{s}_{44}\right)\left({l}_{1}^{2}{l}_{2}^{2}+{l}_{2}^{2}{l}_{3}^{2}+{l}_{3}^{2}{l}_{1}^{2}\right)$$

where ${s}_{{ij}}$ are the elastic compliance constants obtained from the inverse of the stiffness matrix (C_ij_), and *l*_*1*_, *l*_*2*_, *l*_*3*_ are the direction cosines corresponding to the [hkl] orientation.

**Fig. 10:**
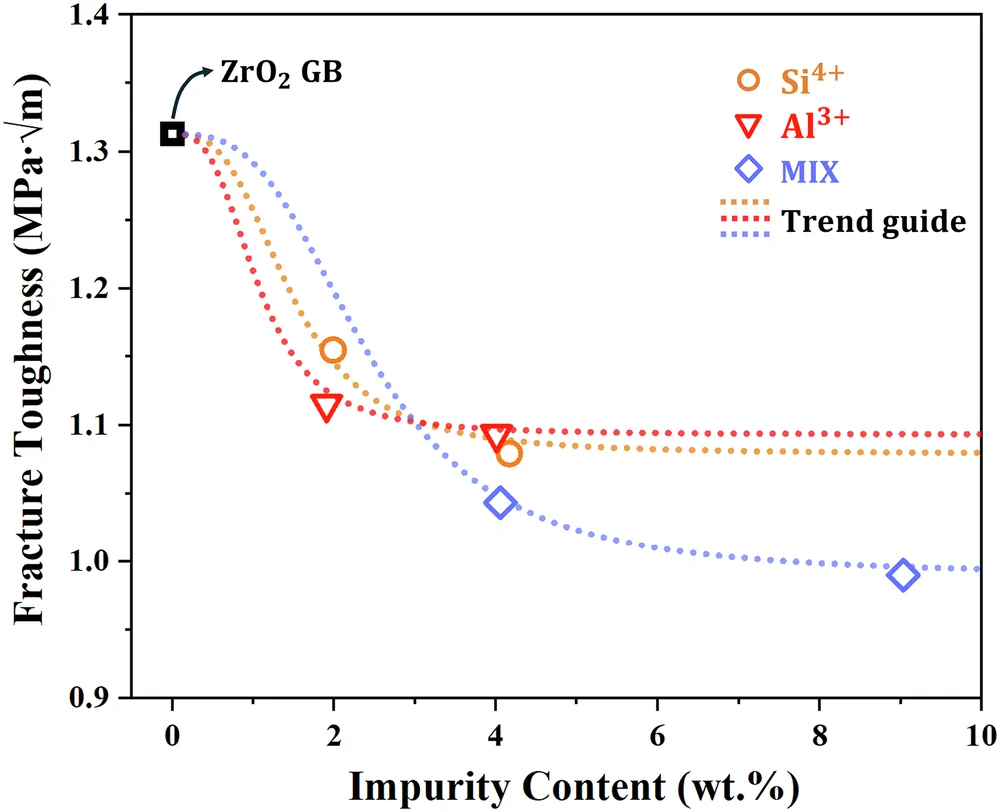
Correlation between CMAS-derived cation content and theoretical GB fracture toughness estimated from separation energy. The orange circles, red triangles, and blue diamonds represent GBs doped by Si^4+^, Al^3+^, and both Si^4+^ and Al^3+^, respectively. The dotted lines are logistic trend guides used only to visualize the calculated trends. The impurity content is defined within the 6.73 Å-wide GB core region used in the DFT model and should not be interpreted as the nominal bulk CMAS content.

Substituting this value into Eq. (1) gives an estimated fracture toughness of 1.312 MPa√m for the clean GB, which is used as the reference value for the representative ZrO₂ GB model in Fig. 10. For the other four models (SI, AL, MIX1 and MIX2), the GB fracture toughness decreased by 12.03%, 15.06%, 17.29%, and 20.52%, respectively. To examine the trend between CMAS-derived impurity content and GB weakening, rather than to establish a predictive quantitative law, we defined a region centered on the site-1 Zr^4+^ with a width of 6.73 Å as the GB core (Fig. 12). The impurity content was defined as the mass fraction of the introduced Si and/or Al cations within this GB core region, rather than the nominal bulk composition of the experimental ceramic. Subsequently, a doubled number of impurity ions were introduced into this region at the same lattice positions (two equivalent sites within the same model). Following the same computational procedures as described above, the theoretical fracture toughness values at this higher concentration were obtained. The corresponding logistic trend guides for each model are presented in Fig. 10. It should be noted that each impurity series contains only the clean GB reference and two doped concentration levels. Therefore, these logistic curves are used only as guides to the eye to visualize the calculated trends and should not be interpreted as predictive quantitative relationships or used for extrapolation. With increasing impurity cation concentration, both single-ion (Si^4+^ or Al^3+^) and mixed-ion doping further embrittle the GB. Interestingly, the mixed-ion case exhibits a more significant effect on GB embrittlement, suggesting a synergistic effect between Si^4+^ and Al^3+^. In addition, the fracture toughness of the GB appears less sensitive to the increase in Al^3+^ concentration than to that of Si^4+^, although the introduction of a small amount of Al^3+^ causes a more noticeable weakening effect. These results indicate that the relative proportions of Al^3+^ and Si^4+^ in CMAS could play an important role in determining the GB fracture toughness. However, additional calculations involving more impurity concentrations, different GB characters, and explicit Y/oxygen-vacancy configurations would be required to establish a fully predictive quantitative model.

Overall, this study demonstrates that CMAS infiltration degrades the grain-boundary structure and fracture resistance of YSZ ceramics through two related pathways. First, CMAS forms nanoscale intergranular glassy films, which provide preferential crack-propagation paths because of their low fracture resistance. Second, CMAS-derived cations, particularly Si⁴⁺ and Al³⁺, can be incorporated into glass-free GBs and weaken GB cohesion even in the absence of a continuous amorphous film. These chemical and structural modifications drive a transition in fracture behavior from predominantly transgranular fracture in pristine YSZ to intergranular fracture in CMAS-affected regions, accompanied by a reduction in local fracture toughness of up to 80%.

The combined experimental and first-principles results show that the degradation of GB fracture resistance is closely linked to the extent and form of intergranular CMAS modification. High-resolution microscopy reveals a depth-dependent transition from CMAS-film-containing GBs near the surface to Si/Al-enriched glass-free GBs at greater depths, while micromechanical testing establishes the corresponding toughness gradient. DFT calculations further indicate that Si⁴⁺ and Al³⁺ incorporation weakens Zr-O bonding along potential fracture paths, increases GB energy and lowers separation energy, providing an atomistic explanation for the observed intergranular embrittlement. These findings highlight the importance of suppressing CMAS penetration and cation segregation along GBs to preserve the mechanical integrity of YSZ-based thermal barrier ceramics.

## Methods

### Materials

**Fig. 11:**
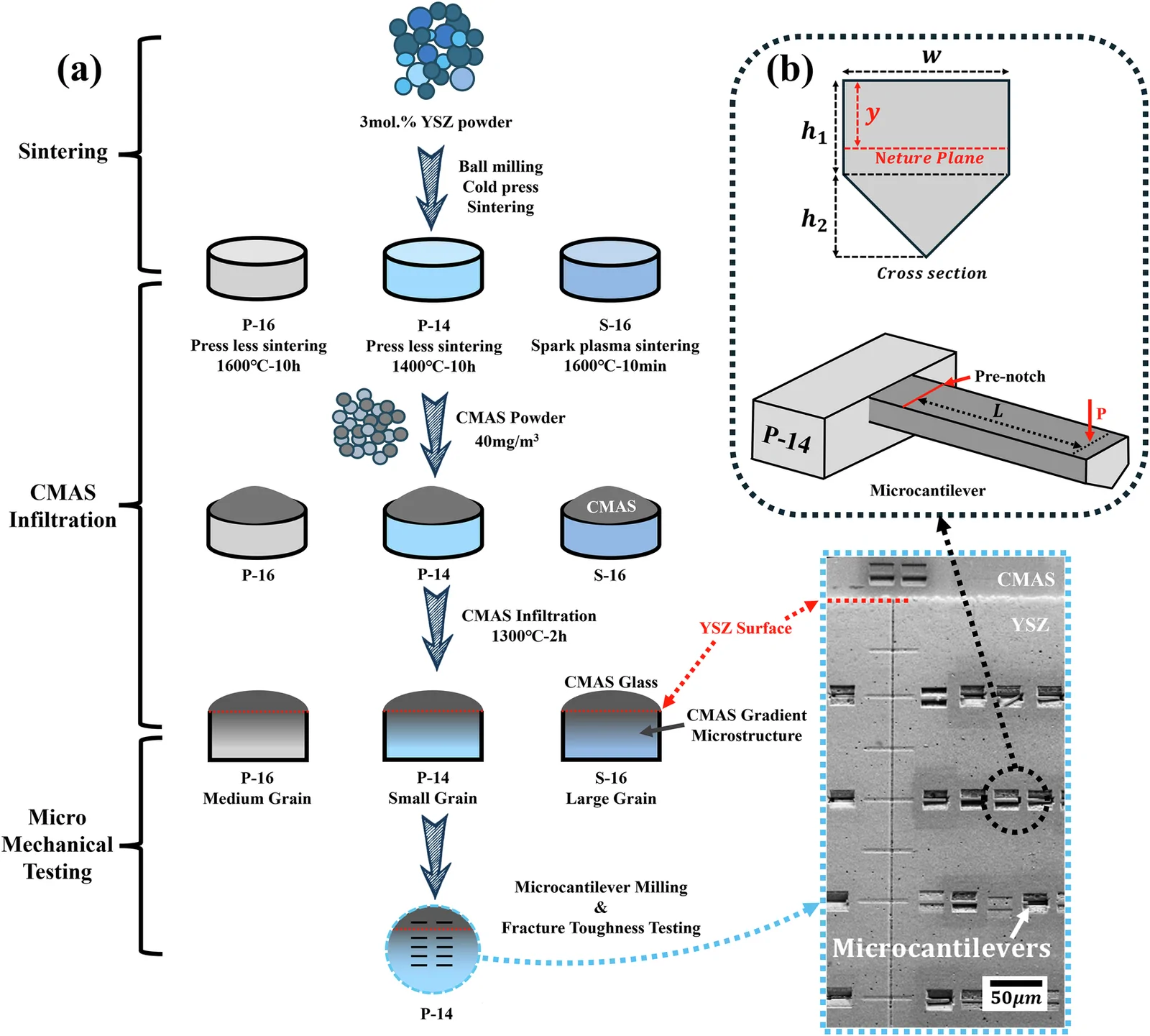
Experimental workflow and schematic description for the preparation of CMAS-infiltrated YSZ ceramics and subsequent micromechanical testing. **a** Preparation procedure of CMAS-infiltrated YSZ ceramics, including infiltration and heat treatment steps. **b** Schematic illustration of the micromechanical testing setup and specimen geometry used for fracture toughness evaluation.

Three types of YSZ pellets with densities above 97% but different grain size were fabricated using different sintering conditions: pressureless sintering at 1400 °C (P-14) and 1600 °C (P-16) for 10 h, and spark plasma sintering at 1600 °C for 10 min under 30 MPa (S-16). The powder used for sintering is 3 mol.% YSZ (PI-KEM, particle size ~0.8 μm) processed by high-energy ball milling in a Fritsch Pulverisette 5 planetary mill for 6 h. After sintering, the ceramics were cut into discs with a diameter of 12 mm and thickness of 5 mm, followed by grinding and polishing. A layer of CMAS (35CaO-10MgO-7AlO_1.5_–48SiO_2_ in mole ratio, supplied by Rolls-Royce) with a dose of 40 mg/cm^2^ was then deposited on the surface of each type of ceramic. The CMAS has a melting point of ~1235 °C ^44^ and was prepared by pre-melting the mixed oxides at 1500 °C for 4 h in a platinum crucible in a CM rapid cycle furnace, followed by air-quenching to room temperature to form homogeneous glass. The CMAS-loaded ceramics were then heat-treated at 1300 °C for 2 h in a Carbolite high-temperature furnace to induce CMAS attack. After cooling, the CMAS-attacked ceramics were cut through along the CMAS infiltration direction and polished to a mirror finish for micromechanical study and characterizations. The experimental workflow for preparation of CMAS-infiltrated YSZ ceramics is schematically illustrated in Fig. 11a.

### Microcantilever beam bending test

Microcantilever beams with pentagonal cross-sections (Fig. 11b) were machined at various depths on the polished cross-sections of CMAS-attacked YSZ ceramics by a focused ion beam (FIB, FEI Helios NanoLab 660) according to the process described in our previous work^25,45–47^. At each depth, five beams were prepared, and their long axes were oriented parallel to the YSZ surface. A sharp notch was introduced approximately 4 μm away from the fixing end of each beam by a small current (80 pA) to guide the crack propagation. The microcantilever beam bending test was conducted in-situ in a scanning electron microscope (SEM, Zeiss, Sigma) using a Hysitron PicoIndenter fitted with a sapphire conical indenter with a tip radius of 2 µm. The bending load was applied to the free end of the beam using a loading rate of 6 µN/s.

The fracture toughness ${K}_{{IC}}$ is given by^48^

3 $${K}_{{IC}}={\sigma }_{c}\sqrt{\pi a}F\left(\frac{a}{h}\right)$$

Where a is the depth of the notch; $F(\frac{a}{h})$ is a dimensionless shape factor dependent on the notch and sample geometry and was computed by finite element analysis based on the procedure outlined previously^49^; h is the height of the beam ($h={h}_{1}+{h}_{2}$, Fig. 11b). ${\sigma }_{C}$ is calculated by

4 $${\sigma }_{c}=\frac{{PLy}}{I}$$

where P is the applied bending force when the beam fails; L is the distance between the notch and the point where the bending force is applied (Fig. 11b); I is the moment of inertia of the beam cross section, and y is the vertical distance between the upper surface and the neutral plane. I and y are given by^49^

5 $$I=\frac{{{wh}}_{1}^{3}}{12}+{\left(y-\frac{{h}_{1}}{2}\right)}^{2}{h}_{1}w+\frac{{{wh}}_{2}^{3}}{36}+{\left[\frac{{h}_{2}}{3}+\left({h}_{1}-y\right)\right]}^{2}\frac{w{h}_{2}}{2}$$

6 $$y=\frac{6{h}_{1}^{2}+3{h}_{1}{h}_{2}+{h}_{2}^{2}}{12{h}_{1}+3{h}_{2}}$$

The meanings of w, h_1_, and h_2_ are illustrated in Fig. 11b.

### Characterizations

The polished cross-sections of the CMAS-attacked ceramics and the fracture surfaces generated from the microcantilever beam bending tests were examined by a SEM (TESCAN MIRA3 LC). SEM images were taken in both secondary electron (SE) and backscattered electron (BSE) mode at 10 kV and 0.8 nA. The GB structure and chemistry of the CMAS-attacked ceramics were studied by a transmission electron microscope (TEM, Talos F200X G2) fitted with energy dispersive X-ray spectroscopy (EDS). TEM lamellae were prepared by FIB milling at different CMAS infiltration depths, extracted in-situ by an Easy-Lift micromanipulator, and welded to a copper grid using a gas injection system. The lamellae were then thinned to an electron-transparent thickness (<100 nm) by progressively reducing the ion beam current for TEM analysis.

### First-principles calculations

As will be demonstrated later, our study reveals that CMAS readily attacks YSZ ceramics through grain boundary infiltration, leading to severe grain boundary embrittlement. An unexpected yet key finding is that YSZ GBs can undergo significant embrittlement through the incorporation of CMAS-derived dopants, such as Al³⁺ and Si⁴⁺, even in the absence of a glassy CMAS film. To reveal the underlying embrittlement mechanisms, first-principles calculations based on DFT were conducted to study the influence of CMAS impurities on the GB bonding and fracture behavior.

**Fig. 12:**
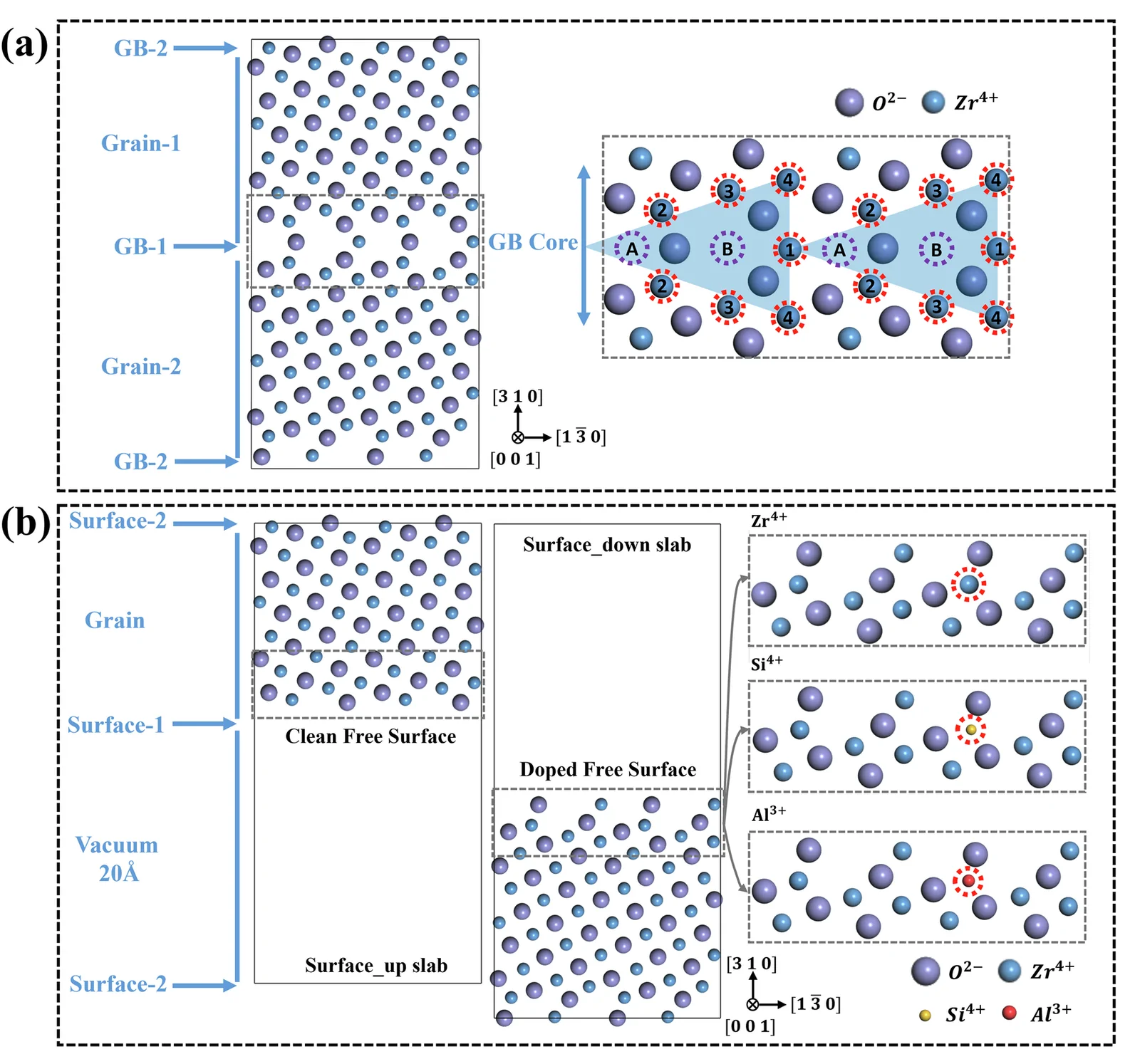
Schematic illustrations of the computational models used for GB and free-surface analyses. **a** The Σ5(310)/[001] ZrO₂ GB supercell, highlighting possible substitutional sites (1-4) and interstitial sites (A, B) for dopant cations derived from CMAS. **b** The ZrO₂ free-surface slab models used for separation-energy calculations, including the surface_down and surface_up slabs, as well as the undoped, Si⁴⁺-substituted, and Al³⁺-substituted surface configurations.

A Σ5(310)/[001] ZrO_2_ model was constructed from a 6 × 3 × 1 ZrO_2_ cubic supercell, comprising 72 Zr^4+^ cations and 144 O^2-^ anions, as shown in Fig. 12a. This boundary was selected due to its favorable symmetry and low energy, allowing the reliable construction of large GB models for first-principles studies^50^. The optimized lattice parameter of the ZrO_2_ super cells is 5.090146 Å, which is consistent with the corresponding bulk experimental values of 5.10 ± 0.010 Å^51^. Two GBs were included in the model to ensure the application of periodic boundary conditions (PBCs). The region with a width of 6.73 Å at the GB center was defined as the GB core, where CMAS cations were introduced either by substituting Zr⁴⁺ ions (sites 1–4) or by insertion into grain boundary voids (sites A and B), as highlighted in (Fig. 12a). For the interstitial cases, to maintain charge balance one neighboring Zr^4+^ ion at the cavity was removed. In the substitutional case, replacing Zr⁴⁺ with trivalent ions (e.g., Al³⁺) introduces a local negative charge in the lattice. This charge imbalance is compensated by the formation of doubly positively charged oxygen vacancies, maintaining overall charge neutrality. Using Kröger-Vink notation, this mechanism can be expressed as:

7 $${2{{Al}}_{2}{O}_{3}+2{{Zr}}_{{Zr}}^{\times }+3{O}_{O}^{\times }\to 2{{Al}}_{{Zr}}+V}_{O}^{\cdot \cdot }+2{{ZrO}}_{2}$$

where ${{Al}}_{{Zr}}$ denotes an ${{Al}}^{3+}$ ion substituting a ${{Zr}}^{4+}$ site, and ${V}_{O}^{\cdot \cdot }$ represents a doubly positively charged oxygen vacancy.

DFT calculations for the above models were performed using the Vienna ab initio Simulation Package (VASP)^52^. Electron-ion interactions were treated with the projector augmented wave (PAW) method^53^ and the exchange-correlation energy was described within the generalized gradient approximation using the Perdew-Burke-Ernzerhof (PBE) functional. The calculations employed PAW pseudopotentials with the following valence configurations: Zr (*4s²4p⁶4d²5* *s²*), O (*2s²2p⁴*), Si (*3s²3p²*), and Al (*3s²3p¹*). PBCs were applied. Structural relaxations were performed using a plane-wave cutoff energy of 450 eV and a 1 × 2 × 6 Monkhorst–Pack k-point mesh to ensure energy convergence. Subsequent single-point calculations employed the same k-point mesh with an increased cutoff energy of 500 eV for improved accuracy. Atomic positions were optimized until the residual forces were below 0.02 eV/Å, and electronic self-consistency was achieved with a threshold of 10⁻⁵ eV. The LOBSTER package (Local Orbital Basis Suite Towards Electronic-Structure Reconstruction)^54^ was used to calculate COHP and ICOHP for the selected supercells, using the self-consistent wavefunctions derived from VASP. Negative COHP values denote bonding interactions, and positive values denote antibonding interactions.

To characterize the bonding behavior and atomic distribution of CMAS elements at GBs, we first calculated the segregation energy of each element at GB core sites described in Fig. 12a to identify their preferred positions. The tendency of a CMAS impurity to accumulate at the GB can be described by its segregation energy, ${E}_{{seg}}^{{GB}}$^55^.

8 $${E}_{{seg}}^{{GB}}=\left({E}_{{tot}}^{{GB}\left[{impurities}\right]}-{E}_{{tot}}^{{GB}\left[{Zr}\right]}\right)-\left({E}_{{tot}}^{{bulk}\left[{impurities}\right]}-{E}_{{tot}}^{{bulk}\left[{Zr}\right]}\right)$$

Where ${E}_{{tot}}^{{GB}}$ and ${E}_{{tot}}^{{bulk}}$ are the lowest total energy without entropy of the GB and bulk models after structure relaxation. The [impurities or Zr] represent that model contains Si, Al or pure ZrO_2_. The bulk reference structure is constructed with identical cell dimensions and atom count as the GB model, but without introducing a boundary^56^. A more negative segregation energy implies greater energetic stability of the model^55^.

Subsequently, based on Griffith’s theory, the GB energy was evaluated as a key parameter for fracture assessment. Higher GB energy indicates that the boundary is less stable and more prone to crack initiation and propagation. From the models described above, the GB energy, γ, can be calculated by,

9 $$\gamma \left[{impurities}/{Zr}\right]=\frac{{E}_{{tot}}^{{GB}\left[{impurities}/{Zr}\right]}-{E}_{{tot}}^{{bulk}\left[{impurities}/{Zr}\right]}}{2A}$$

Where A is the cross-sectional area of the GB plane in the super cell (one model contains two GBs).

Finally, the GB strength was quantified by calculating the separation energy using a surface model, as shown in Fig. 12b. While the GB energy indicates the relative stability of the boundary, the separation energy, E_sep_, represents the energy required to split it into upper and lower free surfaces, reflecting its resistance to fracture, which can be described by,

10 $${E}_{{sep}}=\frac{{E}_{{tot}}^{{surface}{{\_}}{up}}+{E}_{{tot}}^{{surface}{{\_}}{down}}-{E}_{{tot}}^{{GB}}}{2A}$$

Where ${E}_{{tot}}^{{surface\_up}}$ and ${E}_{{tot}}^{{surface\_down}}$ represent the total energies of the surface_up and surface_down slabs on the upper and lower sides, respectively, as illustrated in Fig. 12b. The lower surface-down slab was considered in three configurations: undoped ZrO₂, Si⁴⁺-substituted ZrO₂, and Al³⁺-substituted ZrO₂. Each free-surface slab was constructed from the GB configuration by removing either the upper or lower part of the bicrystal model to expose the corresponding free surface.

## Data Availability

The datasets generated and/or analyzed during the current study are not publicly available due to institutional and industrial-partner data-management policies but are available from the corresponding author on reasonable request.
